# Tumor-suppressive function and mechanism of miR-873-5p in glioblastoma: evidence based on bioinformatics analysis and experimental validation

**DOI:** 10.18632/aging.204800

**Published:** 2023-06-28

**Authors:** Xiaobin Zhang, Fangkun Jing, Chen Guo, Xinning Li, Jianan Li, Guobiao Liang

**Affiliations:** 1 Department of Neurosurgery, General Hospital of the Northern Theater Command of Chinese People’s Liberation Army, Shenyang 110000, China; 2 Department of Neurosurgery, Jinqiu Hospital of Liaoning Province, Shenyang 110000, China

**Keywords:** bioinformatics analysis, glioblastoma, miR-873-5p, HMOX1, HIF1α, SPOP, malignant behaviors

## Abstract

This study aims to clarify the mechanistic actions of microRNA-873-5p (miR-873-5p) on glioblastoma (GBM) progression. The most differentially expressed miRNAs were retrieved from the GEO database. It was established that miR-873-5p was downregulated in GBM tissues and cells. Based on *in silico* prediction and experimental data, HMOX1 was demonstrated to be a target gene of miR-873-5p. Further, miR-873-5p was then ectopically expressed in GBM cells to examine its effect on the malignant behaviors of GBM cells. Overexpression of miR-873-5p inhibited GBM cell proliferation and invasion by targeting HMOX1. HMOX1 promoted SPOP expression by increasing HIF1α expression, thus stimulating GBM cell malignant phenotypes. miR-873-5p suppressed the malignant phenotypes of GBM cells and tumorigenesis *in vitro* and *in vivo* by inhibiting the HMOX1/HIF1α/SPOP signaling axis. This study uncovers a novel miR-873-5p/HMOX1/HIF1α/SPOP axis in GBM, providing new insights into GBM progression and therapeutic targets for GBM treatment.

## INTRODUCTION

Glioblastoma (GBM) represents the most aggressive form of brain tumor [1]. Partly owing to the plastic and heterogenous nature, GBM continues to exhibit very high mortality and morbidity rates [2, 3]. Therefore, gaining a comprehensive understanding of the molecular mechanisms of GBM pathogenesis is urgently needed to develop novel therapeutic strategies for GBM treatment.

Of note, microRNA (miR)-873-5p is a main member of the miR-873 family [4]. A recent study has proved the inhibiting effect of miR-873-5p in glioma cell proliferation and invasion and the promoting effect on cell apoptosis [5]. Meanwhile, miR-873-5p can repress GBM cell growth and migration, but inhibition of miR-873-5p shows opposite effects [6]. miR-873-5p has been demonstrated to target heme oxygenase 1 (HMOX1) and regulate its expression in PC12 cells [7]. HMOX1 is an inducible enzyme capable of catalyzing the degradation of heme [8]. HMOX1 has demonstrated a correlation with the stemness and metastasis of GBM, substantiating the histopathological hallmark of GBM [9]. In human glioma tumors, HMOX1 is robustly induced compared with normal brain tissues and relates to the molecular mechanism driving GBM cell proliferation survival and metastasis [10].

Furthermore, hypoxia-induced factor 1α (HIF1α) transcription is lowered in bone marrow-derived macrophages with HMOX1 knockdown [11]. HIF1α is a subunit of HIF1 activated by hypoxia and a key driver of tumor progression in GBM patients [12, 13]. Elevation of HIF1α promotes the expression of angiogenic factors and elicits vascular abnormality and GBM progression [14]. SPOP encodes an E3 ubiquitin ligase component, and in clear cell renal cell carcinoma, it is a transcriptional target of HIFs [15]. SPOP exerts essential roles in physiological and pathological processes [16]. Notably, a previous study has suggested SPOP as a tumor suppressor in glioma to inhibit cell viability, migration, and invasion *in vitro* [17]. Therefore, it is reasonable to hypothesize that miR-873-5p may serve as a tumor suppressor in GBM with the involvement of HMOX1, HIF1α and SPOP. Consequently, this study employed bioinformatics analysis combined with experimental validation with the aim to validate the role of miR-873-5p in GBM progression and the potential mechanism.

## RESULTS

### miR-873-5p is downregulated in GBM tissues and cell lines, which targets HMOX1

**Figure 1:**
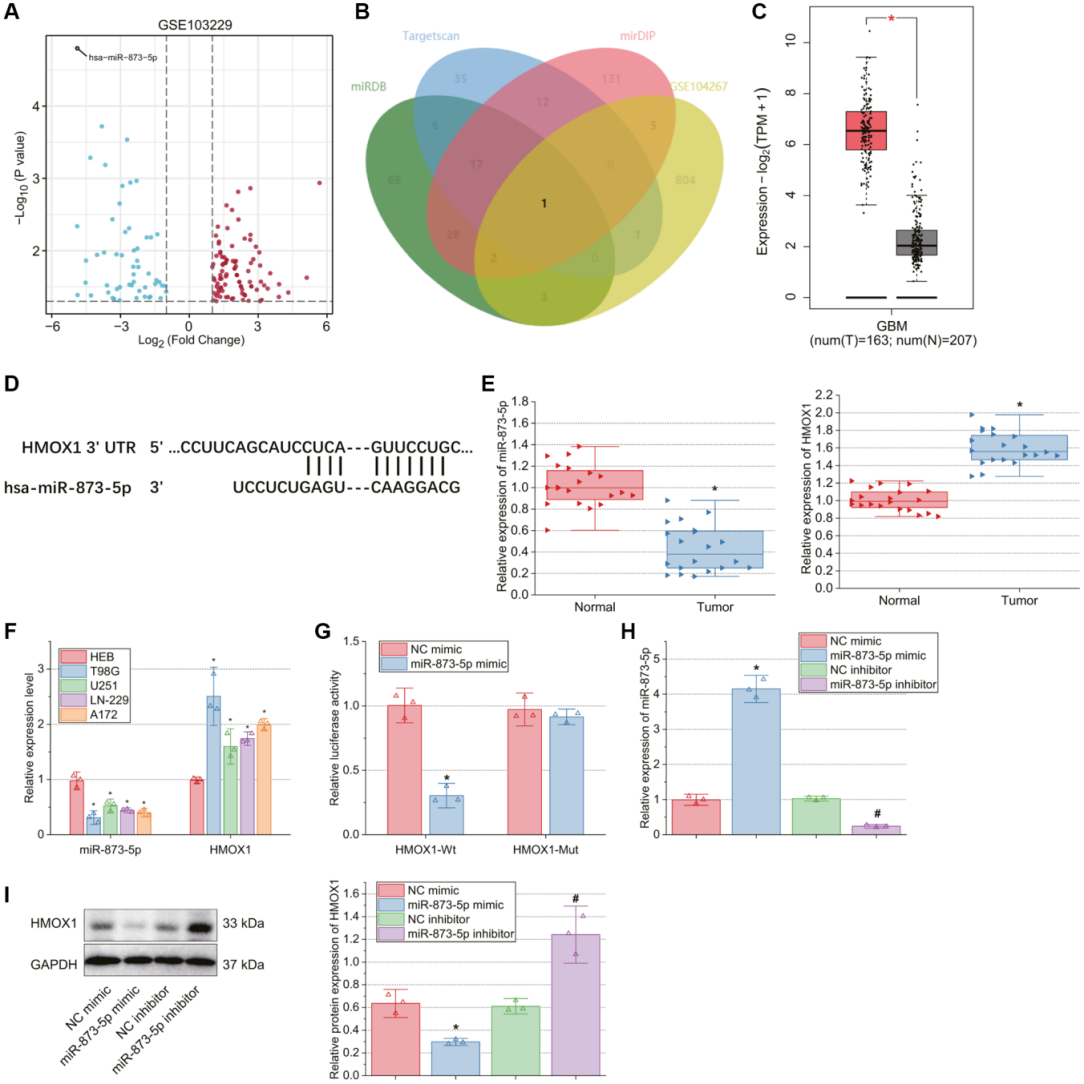
**miR-873-5p, downregulated in GBM tissues or cells, targets HMOX1 and reduces its expression.** (**A**) A volcano plot of the differentially expressed genes in GBM and normal samples from the GSE103229 dataset. Red dots indicate significantly up-regulated genes, and blue dots indicate significantly downregulated genes. The top hsa-miR-873-5p with the most significant differential expression (the lowest *p* value) was labeled. (**B**) Venn diagram of the downstream genes of miR-873-5p predicted using the miRDB, TargetScan, and mirDIP databases with the significantly up-regulated genes in GBM samples from the GSE104267 dataset. (**C**) HMOX1 expression in normal brain tissue samples and GBM samples analyzed by the GEPIA database. The red box represents the GBM tissue samples (*n* = 163), and the gray box represents the normal brain tissue samples (*n* = 207). (**D**) Binding sites of miR-873-5p in HMOX1 mRNA predicted by the miRDB database. (**E**) Expression of miR-873-5p and HMOX1 in tumor tissues of GBM patients (*n* = 20) and normal brain tissues (*n* = 20) measured by RT-qPCR. (**F**) Expression of miR-873-5p and HMOX1 in human normal brain glial cell line HEB and GBM cell lines T98G, U251, LN-229, and A172 determined by RT-qPCR. (**G**) Binding of miR-873-5p to HMOX1 validated by dual-luciferase reporter assay. (**H**) Expression of miR-873-5p in T98G cells transfected with miR-873-5p mimic or miR-873-5p inhibitor determined by RT-qPCR. (**I**) Western blot of HMOX1 protein in T98G cells transfected with miR-873-5p mimic or miR-873-5p inhibitor. ^*^p < 0.05, compared to normal brain tissues, HEB cells or NC mimic-transfected T98G cells. ^#^*p* < 0.05, compared to NC inhibitor-transfected T98G cells.

First, we retrieved a GBM-related miRNA expression dataset, GSE103229, from the GEO database and conducted the differential analysis with |logFC| > 1 and *p* < 0.05 as the threshold. miR-873-5p was found to be the most significantly differentially expressed miRNA, with a significant down-regulation in brain tissue samples from GBM patients compared to normal brain tissue samples (Figure 1A).

miRDB, TargetScan, and mirDIP databases predicted 123, 72 and 196 downstream target genes of miR-873-5p, respectively. Meanwhile, 816 significantly up-regulated genes were obtained following the differential analysis of the GBM-related gene expression dataset GSE104267, and then intersected with the predicted downstream genes, with one candidate gene determined, namely HMOX1 (Figure 1B). According to the analysis results of the GEPIA database, HMOX1 expression was amplified in GBM samples relative to normal brain tissue samples (Figure 1C). In addition, miR-873-5p binding sites in HMOX1 mRNA were found following analysis using the miRDB database (Figure 1D).

Based on RT-qPCR results, miR-873-5p expression was lowered while HMOX1 expression was elevated in tumor tissues of GBM patients compared with normal tissues (Figure 1E). Furthermore, similar results were obtained in the T98G, U251, LN-229, and A172 cells compared to HEB cells. Of note, the T98G cell line showed the lowest miR-873-5p expression and the highest HMOX1 expression (Figure 1F), which was thus selected for subsequent experiments.

Dual-luciferase reporter assay results demonstrated a decline of HMOX1-Wt luciferase activity upon miR-873-5p mimic transfection, while no changes were noted in HMOX1-Mut luciferase activity (Figure 1G), indicating that miR-873-5p could bind to HMOX1.

To test miR-873-5p regulation on HMOX1 expression, we transfected T98G cells with miR-873-5p mimic and miR-873-5p inhibitor. RT-qPCR determined the transfection efficiency (Figure 1H). In addition, western blot results showed reduced HMOX1 expression in T98G cells with miR-873-5p mimic, while opposite results were found upon miR-873-5p inhibitor (Figure 1I).

### miR-873-5p inhibits GBM cell malignant characteristics via targeting HMOX1

**Figure 2:**
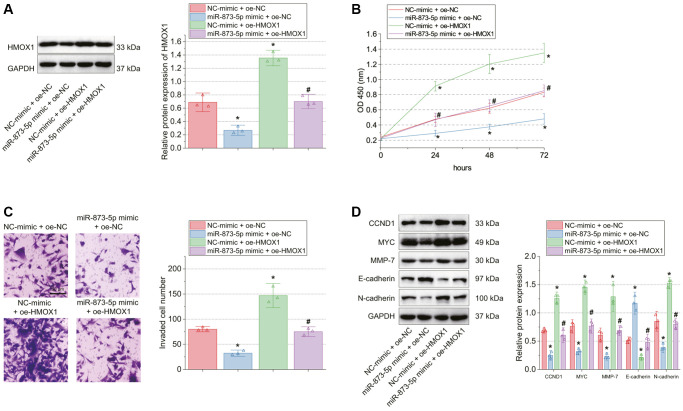
**miR-873-5p impairs GBM cell proliferation and invasion by targeting HMOX1.** T98G cells were transfected with miR-873-5p mimic, oe-HMOX1 or in combination. (**A**) Western blot of HMOX1 protein in T98G cells. (**B**) Proliferation of T98G cells measured by CCK-8 assay. (**C**) Invasion of T98G cells measured by Transwell assay. (**D**) Western blot of the CCND1, E-cadherin, MYC, MMP-7, and N-cadherin proteins in T98G cells. ^*^p < 0.05, compared to NC-mimic + oe-NC-transfected T98G cells. ^#^*p* < 0.05, compared to miR-873-5p mimic + oe-NC-transfected T98G cells. The cell experiment was run in triplicate independently.

Then, we shifted to testing the effect of miR-873-5p and HMOX1 on GBM cell functions. HMOX1 protein expression was decreased in T98G cells transfected with miR-873-5p mimic + oe-NC while it was increased in response to oe-HMOX1 alone or combined with miR-873-5p mimic (Figure 2A).

Moreover, a reduction of cell proliferation and invasion was noted upon miR-873-5p overexpression, the effect of which was undermined by HMOX1 overexpression (Figure 2B, 2C). Meanwhile, the protein expression of CCND1, MYC, MMP-7, and N-cadherin was reduced while that of E-cadherin was increased in T98G cells with miR-873-5p mimic + oe-NC; conversely, oe-HMOX1 alone or combined with miR-873-5p mimic caused opposite results (Figure 2D).

### HMOX1 up-regulates SPOP expression by augmenting HIF1α expression

**Figure 3:**
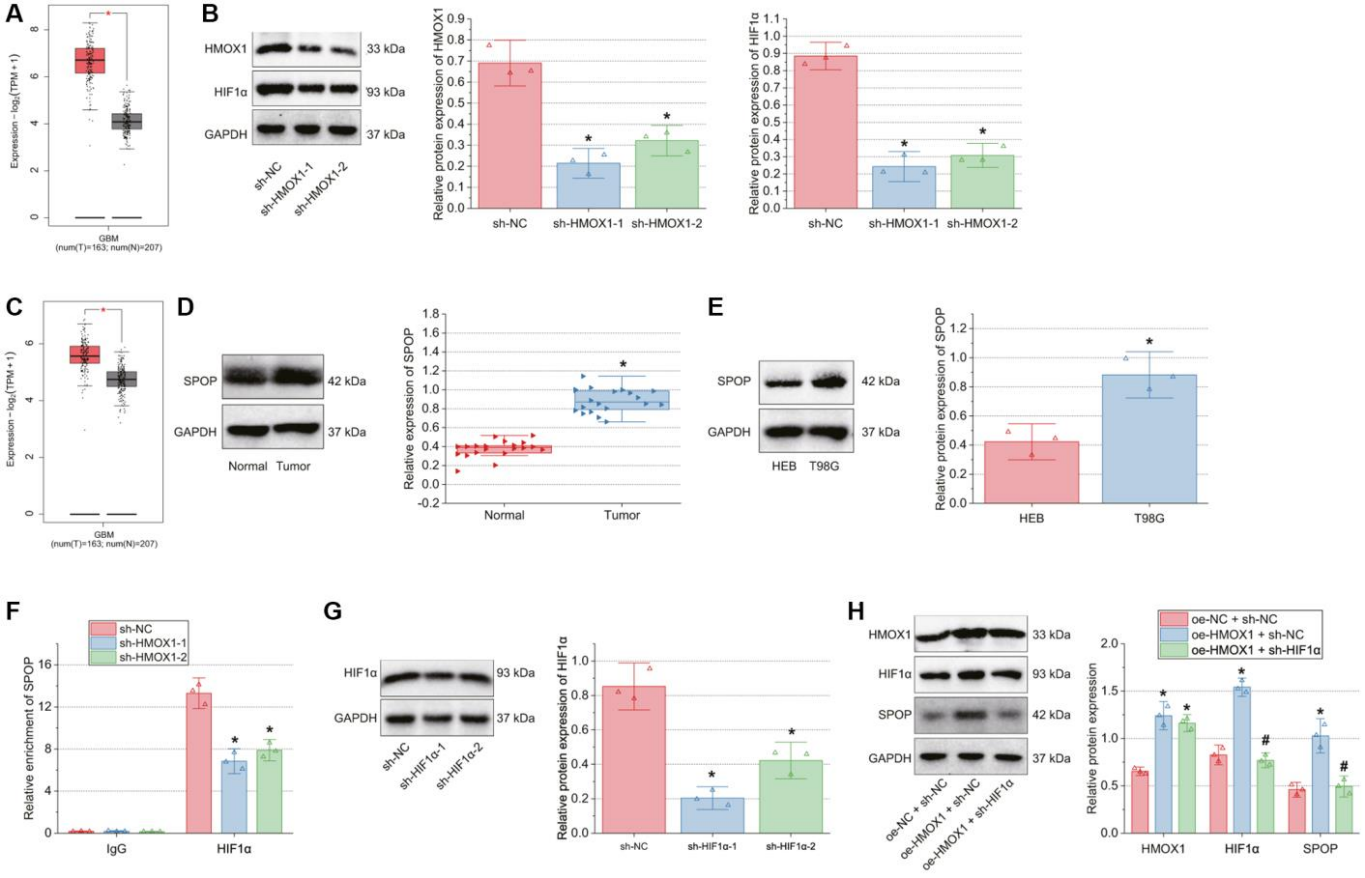
**HMOX1 up-regulates SPOP expression by promoting HIF1α expression.** (**A**) Expression of HIF1α in normal brain tissue samples and GBM tissue samples analyzed by the GEPIA database. The red box represents the GBM tissue samples (*n* = 163) and the gray box represents the normal brain tissue samples (*n* = 207). (**B**) Transfection efficiency of sh-HMOX1 in T98G cells determined by Western blot. (**C**) Expression of SPOP in normal brain tissue samples and GBM tissue samples analyzed by the GEPIA database. The red box represents the GBM tissue samples (*n* = 163), and the gray box represents the normal brain tissue samples (*n* = 207). (**D**) Western blot of SPOP protein in tumor tissues of GBM patients (*n* = 20) and normal brain tissues (*n* = 20). (**E**) Western blot of SPOP protein in HEB and T98G cells. (**F**) Enrichment of SPOP promoter using anti-HIF1α antibody detected by ChIP assay. (**G**) Transfection efficiency of sh-HIF1α in T98G cells determined by Western blot. (**H**) Western blot of HMOX1, HIF1α and SPOP proteins in T98G cells transfected with oe-HMOX1 alone or combined with sh-HIF1α. ^*^p < 0.05, compared to normal brain tissues, HEB cells or T98G cells transfected with sh-NC or oe-NC + sh-NC. ^#^*p* < 0.05, compared to oe-HMOX1 + sh-NC-transfected T98G cells. The cell experiment was run in triplicate independently.

Analysis of the GEPIA database indicated that HIF1α expression was augmented in the GBM samples versus the normal brain tissue samples (Figure 3A). In addition, Western blot results showed reduced expression of HMOX1 and HIF1α in T98G cells treated with sh-HMOX1 (Figure 3B).

Previous literature has reported that HIF1α can transcriptionally activate the expression of SPOP [15], which has tumor-promoting effects in several types of cancer [15, 18, 19]. Here, we found that SPOP was significantly up-regulated in GBM samples by GEPIA analysis (Figure 3C). Meanwhile, SPOP protein expression was amplified in tumor tissues of GBM patients (Figure 3D), and in T98G cells (Figure 3E) compared with normal tissues and HEB cells, respectively.

We observed decreased enrichment of SPOP promoter in ChIP assays using specific antibody for HIF1α (Figure 3F). Furthermore, Western blot verified the transfection efficiency of sh-HIF1α in T98G cells, as shown by reduced HIF1α protein expression in T98G cells transfected with sh-HIF1α-1 or sh-HIF1α-2. The sh-HIF1α-1 sequence showed better efficiency (Figure 3G) and was used for the subsequent experiments.

In response to oe-HMOX1 + sh-NC transfection, HMOX1, HIF1α, and SPOP protein expression was increased, while further sh-HIF1α transfection resulted in decreased protein expression of HIF1α and SPOP without affecting that of HMOX1 (Figure 3H).

### HMOX1 promotes GBM cell malignant characteristics through activation of the HIF1α/SPOP signaling axis

**Figure 4:**
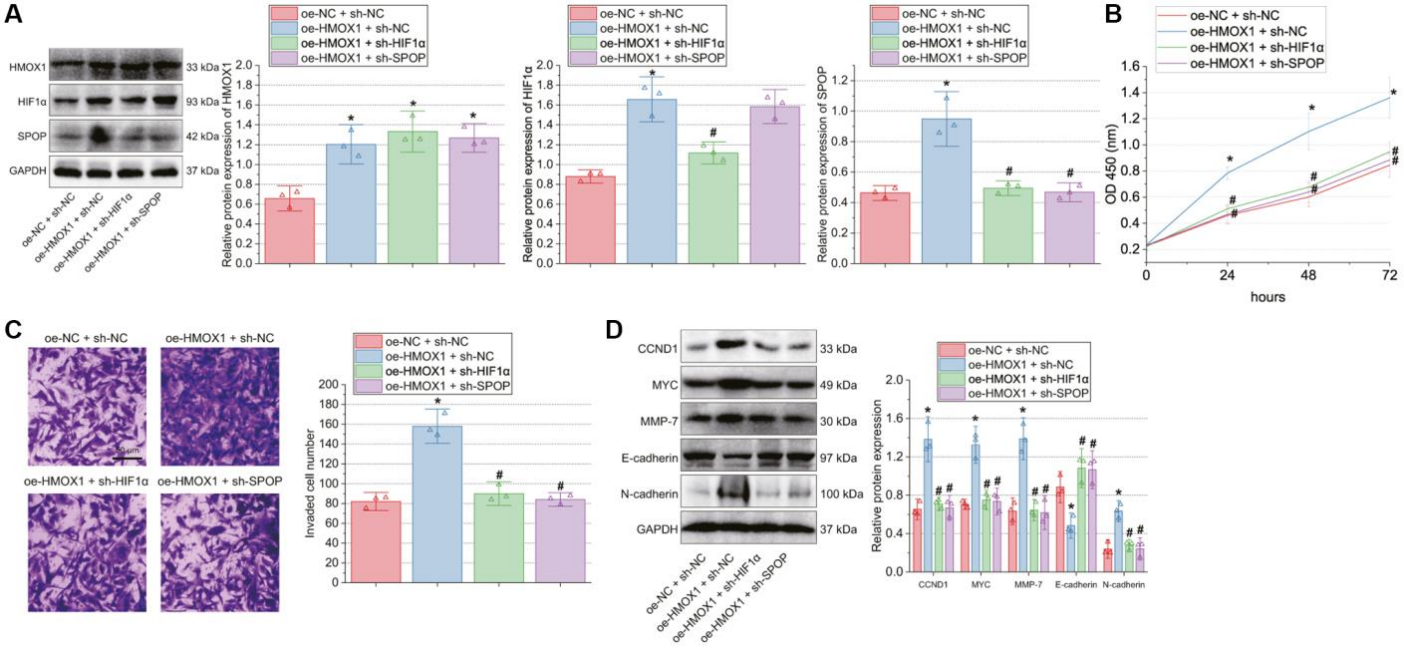
**HMOX1 promotes GBM cell proliferation and invasion through activation of the HIF1α/SPOP signaling axis.** T98G cells were transfected with oe-HMOX1, oe-HMOX1 + sh-HIF1α or oe-HMOX1 + sh-SPOP. (**A**) Western blot of HMOX1, HIF1α and SPOP proteins in T98G cells. (**B**) Proliferation of T98G cells measured by CCK-8 assay. (**C**) Invasion of T98G cells measured by Transwell assay. (**D**) Western blot of the CCND1, E-cadherin, MYC, MMP-7, and N-cadherin proteins in T98G cells. ^*^p < 0.05, compared to oe-NC + sh-NC-transfected T98G cells. ^#^*p* < 0.05, compared to oe-HMOX1 + sh-NC-transfected T98G cells. The cell experiment was run in triplicate independently.

Next, this study focused on the effect of HMOX1 on GBM cell function by regulating the HIF1α/SPOP signaling axis. Western blot results presented increased expression of HMOX1, HIF1α, and SPOP in the T98G cells transfected with oe-HMOX1 + sh-NC while further transfection with sh-HIF1α reduced HIF1α and SPOP expression. Upon transfection with oe-HMOX1 + sh-SPOP, HMOX1 and HIF1α expression was unchanged in addition to diminished SPOP expression (Figure 4A).

As shown in Figure 4B, 4C, cell proliferation and invasion were augmented upon HMOX1 overexpression, whereas contrary results were found following HIF1α silencing or SPOP silencing. Meanwhile, HMOX1 overexpression up-regulated CCND1, MYC, MMP-7 and N-cadherin protein expression while downregulating E-cadherin protein expression; in contrast, HIF1α silencing or SPOP silencing abolished these effects of HMOX1 overexpression (Figure 4D).

### miR-873-5p inhibits GBM cell malignant characteristics by disrupting the HMOX1/HIF1α/SPOP signaling axis

**Figure 5:**
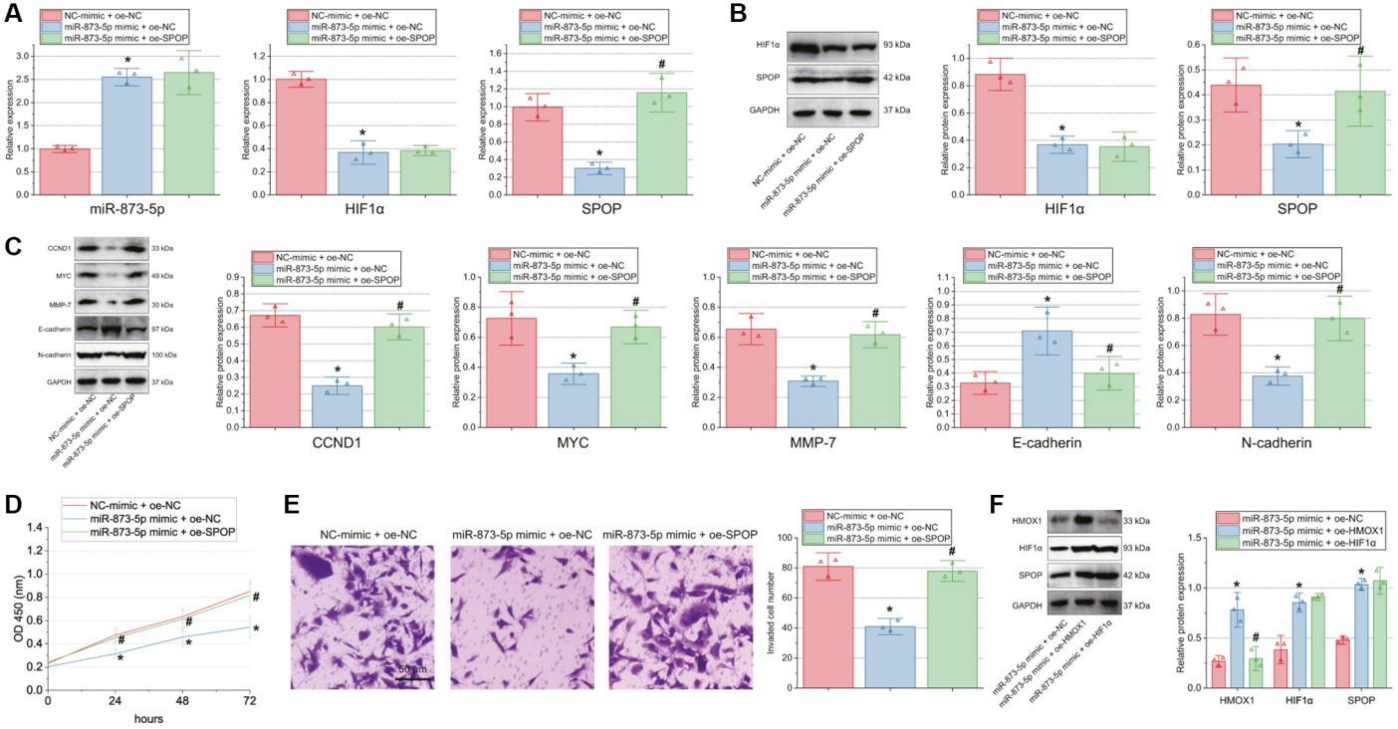
**miR-873-5p inhibits GBM cell proliferation and invasion by inhibiting the HMOX1/HIF1α/SPOP signaling axis.** T98G cells were transfected with miR-873-5p mimic alone or combined with oe-SPOP. (**A**) Expression of miR-873-5p, HIF1α and SPOP in T98G cells determined by RT-qPCR. (**B**) Western blot of HIF1α and SPOP proteins in T98G cells. (**C**) Western blot of the CCND1, E-cadherin, MYC, MMP-7, and N-cadherin proteins in T98G cells. (**D**) Proliferation of T98G cells measured by CCK-8 assay. (**E**) Invasion of T98G cells measured by Transwell assay. (**F**) Western blot of HMOX1, HIF1α, and SPOP proteins in T98G cells transfected with miR-873-5p mimic alone or combined with oe-HMOX1 or oe-HIF1α. ^*^p < 0.05, compared to NC-mimic + oe-NC-transfected T98G cells. ^#^*p* < 0.05, compared to miR-873-5p mimic + oe-NC-transfected T98G cells. The cell experiment was run in triplicate independently.

The aforementioned results allowed us to determine whether miR-873-5p affected GBM cell function through the HMOX1/HIF1α/SPOP signaling. Transfection with miR-873-5p mimic + oe-NC in T98G cells amplified miR-873-5p expression and reduced the expression of HIF1α and SPOP. Further transfection with oe-SPOP failed to affect miR-873-5p and HIF1α expression but elevated SPOP expression (Figure 5A, 5B).

Furthermore, Western blot data exhibited a decline of CCND1, MYC, MMP-7, and N-cadherin expression, yet an increase of E-cadherin expression upon abundantly expressed miR-873-5p. However, oe-SPOP alone or combined with miR-873-5p mimic led to contrasting results (Figure 5C). In addition, decreased cell proliferation and invasion were noted upon miR-873-5p overexpression, the effect of which was rescued by oe-SPOP alone or combined with miR-873-5p mimic (Figure 5D, 5E). To further confirm that miR-873-5p regulates SPOP expression via HMOX1/HIF1α, we transfected T98G cells with miR-873-5p mimic + oe-NC, miR-873-5p mimic + oe-HMOX1 and miR-873-5p mimic +oe-HIF1α. The results of Western blot suggested higher expression of HMOX1, HIF1α and SPOP in T98G cells transfected with miR-873-5p mimic + oe-HMOX1 than those with miR-873-5p mimic + oe-NC. In addition, transfection with miR-873-5p mimic + oe-HIF1α did not change the expression of HMOX1 while augmenting that of HIF1α and SPOP versus transfection with miR-873-5p mimic + oe-NC (Figure 5F).

Overall, miR-873-5p can repress GBM cell malignant characteristics by inhibiting the HMOX1/HIF1α/SPOP signaling axis.

### miR-873-5p curtails the tumorigenesis of GBM cells by inhibiting the HMOX1/HIF1α/SPOP signaling axis *in vivo*

**Figure 6:**
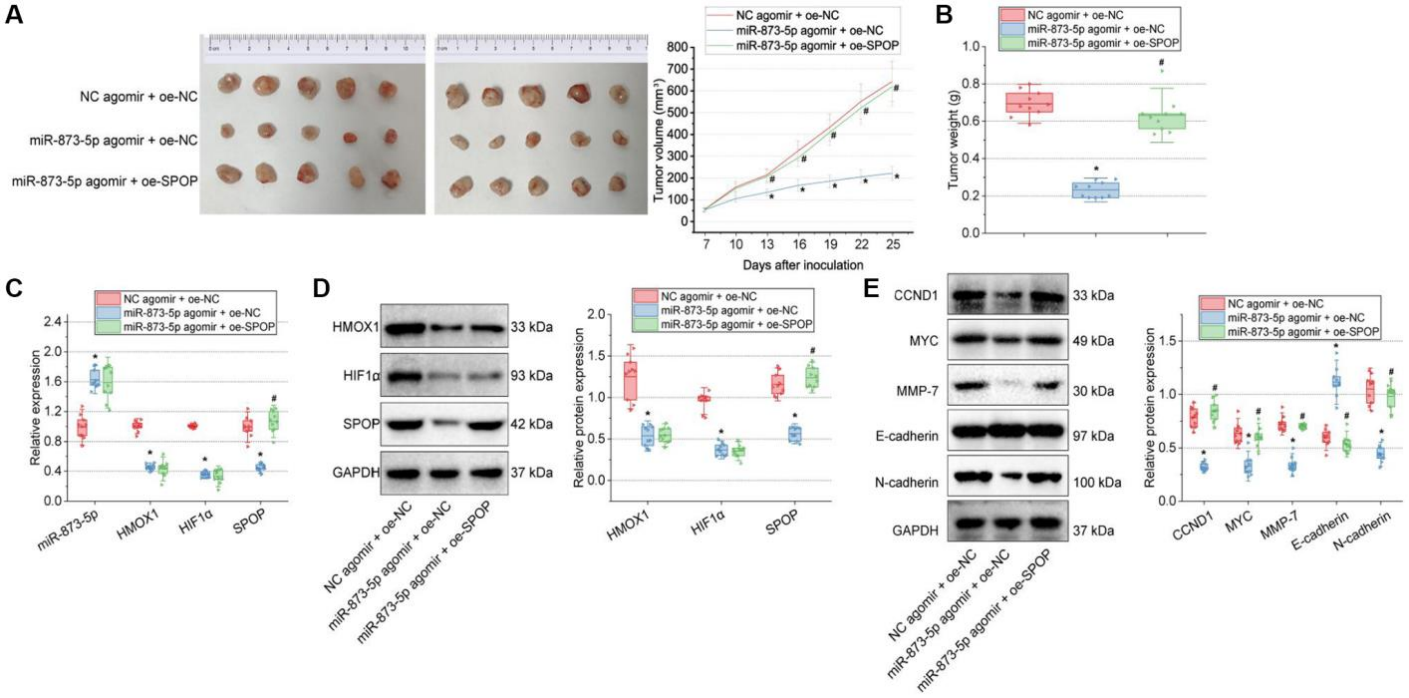
**miR-873-5p inhibits the tumorigenesis of GBM cells by downregulating the HMOX1/HIF1α/SPOP signaling axis *in vivo*.** (**A**) Tumor volume of nude mice. (**B**) Tumor weight of nude mice. (**C**) Expression of miR-873-5p, HMOX1, HIF1α, and SPOP in the tumor tissues of mice detected by RT-qPCR. (**D**) Western blot of the HMOX1, HIF1α, and SPOP proteins in the tumor tissues of mice. (**E**) Western blot of the CCND1, E-cadherin, MYC, MMP-7, and N-cadherin proteins in the tumor tissues of mice. *n* = 10. ^*^p < 0.05, compared to NC agomir-treated mice. ^#^*p* < 0.05, compared to miR-873-5p agomir + oe-NC-treated mice.

Finally, the effect of miR-873-5p on the tumorigenesis of GBM cells *in vivo* was tested in the *in vivo* animal experiments. Injection of miR-873-5p agomir + oe-NC-treated T98G cells into the nude mice decreased tumor volume and weight, while miR-873-5p agomir + oe-SPOP led to opposite results (Figure 6A, 6B).

Furthermore, expression of miR-873-5p was increased, but that of HMOX1, HIF1α, and SPOP was reduced in the tumor tissue of mice treated with miR-873-5p agomir + oe-NC. However, further treatment with oe-SPOP caused no changes in miR-873-5p, HMOX1, and HIF1α expression and enhanced SPOP expression (Figure 6C, 6D).

A decline of CCND1, MYC, MMP-7, and N-cadherin expression along with an increase of E-cadherin expression was evident following treatment with miR-873-5p agomir + oe-NC; miR-873-5p agomir + oe-SPOP contributed to contrasting results (Figure 6E). Collectively, miR-873-5p can target HMOX1 and consequently downregulate the HIF1α/SPOP signaling axis, delaying the tumorigenesis of GBM cells *in vivo*.

## DISCUSSION

This study revealed that miR-873-5p can target HMOX1 and reduce its expression, and leads to consequent downregulation of the HIF1α/SPOP signaling axis, ultimately suppressing GBM cell growth and tumorigenesis, based on bioinformatics analysis together with experimental validation.

The results of this study showed that miR-873-5p was poorly expressed in GBM tissues and cell lines; in addition, the abundant expression of miR-873-5p could repress the malignant functions of GBM cells *in vitro* along with cell tumorigenesis *in vivo*. Indeed, miR-873-5p has been found to be down-regulated in GBM tissues; and overexpression of miR-873-5p exerts tumor-inhibiting roles to arrest the growth and migration of GBM cells [20]. Additionally, miR-873 has been observed to suppress the expression of IGF2BP1 and inhibit GBM cell tumorigenesis and metastasis [21].

Subsequent results showed that the inhibiting effect of miR-873-5p on GBM cell malignant behaviors was associated with the reduction of the miR-873-5p target gene HMOX1. In line with this finding, a previous study has demonstrated that miR-873-5p can target HMOX1 and negatively regulate its expression in PC12 cells [7]. HMOX1 is obviously up-regulated in GBM cells and tissues and can serve as a potential biopsy marker for GBM [22, 23]. In addition, evidence has shown that the knockdown of HMOX1 contributes to the impairment of GBM invasion [24]. Thus, miR-873-5p-mediated inhibition of HMOX1 may represent a useful target for inhibiting GBM tumor progression.

Notably, the present study revealed the possible downstream mechanism of HMOX1 promoting GBM cell proliferation and invasion, which was due to the activation of the HIF1α/SPOP signaling. In specific terms, HMOX1 promoted SPOP expression by increasing HIF1α expression and thus stimulated the malignant phenotypes of GBM cells. HMOX1 acts as upstream of HIF1α and can promote the stabilization of HIF1α [8]. Previous data indicate that SPOP protein can be regulated by HIF1α at the post-transcription level and accumulation of HIF1α augments the expression of SPOP in trophoblasts [25]. Meanwhile, amplified expression of HIF1α was noted in GBM brain tissues and cell lines; diminishing the expression of HIF1α leads to a decrease in U87MG cell viability [26]. In addition, inhibition of HIF1α can retard tumor development in a murine U251-xenograft model of GBM [27]. Another study has highlighted that the knockout of HIF1α in GBM cells eradicates cell invasion *in vitro* and inhibits tumor growth *in vivo* [28]. SPOP can suppress tumorigenesis in various human malignancies, including lung, prostate, colon, gastric, and liver cancers; however, there is also evidence revealing that SPOP exhibits oncogenic functions in kidney cancer [18]. The possible function of SPOP in GBM, however, has not yet been reported. Further investigation is still required to validate the promoting property of SPOP on GBM cell malignant phenotype along with tumorigenesis *in vivo*.

In addition to the above results, the current research also found that ectopically expressed miR-873-5p or HIF1α silencing could reduce the expression of N-cadherin while increasing that of E-cadherin in T98G cells. Conversely, overexpression of HMOX1 or SPOP led to opposite results. E-cadherin and N-cadherin are proteins related to EMT, which is critical in tumorigenesis due to its enhancement in chemoresistance, metastasis, and tumor stemness. Moreover, the up-regulation of N-cadherin and the down-regulation of E-cadherin are the hallmarks of EMT [29, 30]. Furthermore, existing evidence has shown that miR-873-5p overexpression can inhibit colorectal cancer cell migration, invasion, and EMT formation [31]. Another study also showed that miR-873-5p suppresses EMT and colorectal cancer cell proliferation via directly targeting JMJD8 through the NF-κB pathway [32]. In addition, HMOX1 expression has been found to significantly enhance EMT and promote lung adenocarcinoma metastasis [33]. In clear cell renal cell carcinoma, SPOP drives EMT and promotes cell invasion via activation of β-catenin/TCF4 complex [34]. Mounting studies have demonstrated that the EMT of cancer cells can be enhanced by overexpression of HIF1α [35, 36]. Therefore, miR-873-5p may contribute to the suppression of GBM EMT and invasiveness by downregulating the HMOX1/HIF1α/SPOP signaling axis.

**Figure 7:**
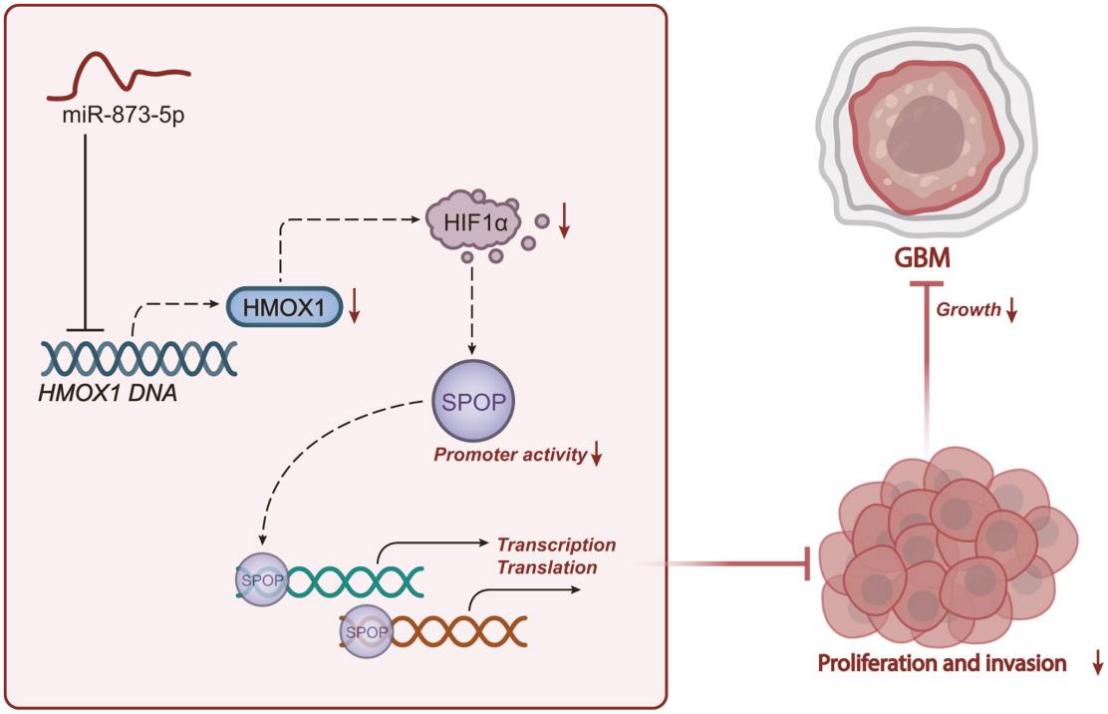
**Schematic representation of the mechanism of miR-873-5p in GBM.** miR-873-5p targets HMOX1 and inhibits its expression, thus downregulating the HIF1α/SPOP signaling axis, reducing the proliferation and invasion as well as the tumorigenesis of GBM cells.

In conclusion, the present study suggested that miR-873-5p may act as a tumor suppressor in GBM progression by downregulating the HMOX1/HIF1α/SPOP signaling axis (Figure 7). These findings provide a mechanistic understanding for developing alternative strategies for GBM treatment. Nonetheless, HIF1α is directly related to the hypoxia environment and signaling pathways [37, 38], and SPOP is a commonly studied E3 ubiquitin ligase [18, 39]. Meanwhile, another study has revealed that hypoxia-induced GLT8D1 can impede CD133 degradation via N-linked glycosylation. Direct blockade of the GLT8D1/CD133 complex formation through CD133N1-108, or inhibiting GLT8D1 expression through lercanidipine can suppress Wnt/β-catenin-dependent tumorigenesis *in vitro* and in a xenograft mouse model of GBM patient-derived tumors [40]. In addition, ivacaftor has been identified as an effective inhibitor of GBM stem cell maintenance, which can promote cell apoptosis and delay the growth of patient-derived xenografts from GBM. Besides, ivacaftor reduces the expression of stem cell marker genes, including CD133, CD44, and Sox2, thereby inhibiting the progression of GBM [41]. Therefore, in the context of GBM, the involvement of other hypoxia-related pathways cannot be excluded in the pronounced protection of miR-873-5p against GBM.

## MATERIALS AND METHODS

### *In silico* prediction

GBM-related miRNA expression dataset GSE103229 and mRNA expression dataset GSE104267 were retrieved from the GEO database. The GSE103229 dataset contains 5 normal brain tissue samples and 5 brain tissue samples from GBM patients. The GSE104267 dataset contains 3 control brain tissue samples and 9 brain tissue samples from GBM patients. Differentially expressed miRNAs and genes were screened using R “limma” package, and |logFC| > 1 and *p* value < 0.05 served as the threshold.

Downstream target genes of miR-873-5p were predicted using miRDB (Target Score > 80), TargetScan (Total context++ score < −0.20), and mirDIP databases (Score Class = Very High). The predicted genes underwent intersection analysis with the DEGs from the GSE104267 dataset. The GEPIA database was used to analyze HMOX1, HIF1α, and SPOP expression in normal brain tissue samples (*n* = 207) and GBM tissue samples (*n* = 163).

### Tissue sample collection

Tumor tissue samples were collected from 20 patients with GBM receiving tumor resection between August 2020 and August 2021. All patients (aged 20-68 years with a mean age of 46 years; 12 males and 8 females) had no anti-tumor treatment before surgery. The normal brain tissues of 20 cases of accidental injuries requiring brain surgery were used as the control. No patients underwent chemotherapy or radiotherapy before surgery. The included patients were confirmed as GBM as per the 2016 WHO Classification of Tumors of the Central Nervous System and pathological examination report. Tissue specimens were stored in liquid nitrogen.

### Cell culture and treatment

Human normal brain glial cell line HEB (27142244, Shanghai Aolu Biotechnology Co. Ltd., Shanghai, China) was cultured with DMEM (11965092, Gibco; consisted of 100 U/mL penicillin, 100 μg/mL streptomycin, 10% FBS). In addition, GBM cell lines (Procell Life Science and Technology Co. Ltd., Wuhan, China) T98G (CL-0583), U251 (CL-0237), LN-229 (CL-0578) and A172 (CL-0012) were cultured with EMEM (30-2003, ATCC, Manassas, VA, USA; encompassing 100 U/mL penicillin, 100 μg/mL streptomycin, 10% FBS). Cells were cultured at 37°C, 5% CO_2,_ and 95% saturated humidity [42].

Using Lipofectamine 2000 reagent (11668019, Invitrogen), T98G cells were transfected with plasmids (GenePharma, Shanghai, China) of miR-873-5p inhibitor, miR-873-5p mimic, NC mimic, NC inhibitor, NC-mimic + oe-NC, miR-873-5p mimic + oe-NC, NC-mimic + oe-HMOX1, miR-873-5p mimic + oe-HMOX1, miR-873-5p mimic + oe-HIF1α, sh-NC, sh-HMOX1, oe-HMOX1 + sh-NC, oe-HMOX1 + sh-HIF1α and oe-HMOX1 + sh-SPOP. The transfection sequences are described in Supplementary Table 1.

### RT-qPCR and Western blot

Total RNA was extracted by TRIzol (15596026, Invitrogen, USA) and reverse transcribed into cDNA by Tailing method using EZ-press miRNA Reverse Transcription Kit (EZB-miRT2-S, for miRNA detection; EZBioscience, USA) or PrimeScript RT reagent Kit (Takara, Japan; RR047A, for mRNA detection). ABI 7500 quantitative PCR instrument (Applied Biosystems, Waltham, MA, USA) was applied for RT-qPCR. 2^−ΔΔCT^ was employed to calculate levels of target genes, normalized to U6 or GAPDH. Primer sequences are shown in Supplementary Table 2.

Total protein was extracted, separated by SDS-PAGE, and transferred onto PVDF membranes. The membrane was blocked with 5% skim milk powder and probed overnight at 4°C with primary rabbit antibodies (Supplementary Table 3) and then with secondary antibody (ab97051, 1: 2000, Abcam, HRP-labeled goat anti-rabbit IgG H&L). ECL reagent (Amersham, Little Chalfont, UK, BB-3501) was employed to visualize protein bands. Quantity One v4.6.2 software was applied for band intensity quantification, normalized to GAPDH [43].

### Dual-luciferase reporter assay

The cDNA fragment of the HMOX1 3′-UTR containing miR-873-5p binding sites was cloned into the pmirGLO vector. Through the site-directed mutagenesis, the HMOX1 3′-UTR fragment (HMOX1-Mut) was cloned into the pmirGLO vector. The two plasmids were transfected with miR-873-5p mimic or NC mimic into HEK293T cells (CL-0005, Procell; cultured with DMEM containing 10% FBS at 37°C and 5% CO_2_) for 48 h. Next, luciferase activities were detected by a multi-mode microplate reader (SpectraMax M5, Molecular Devices, Sunnyvale, CA, USA).

### CCK-8 assay

CCK-8 kit (CK04, Dojindo Laboratories, Kumamoto, Japan) was used for this experiment. GBM cells were seeded into 96-well plates (1 × 10^4^ cells/well) and cultured for 24 h. Then cells were incubated at 37°C for 3 h with CCK-8 reagent (10 μL). OD values at 450 nm were determined.

### Transwell assay

The 50 μL of Matrigel (YB356234, Yubo Biotechnology Co. Ltd., Shanghai, China) was spread in each Transwell chamber. The cells were digested, counted, and resuspended. The upper chamber was added with 200 μL cell suspension, and 800 μL conditioned medium with 20% FBS was supplemented to the lower chamber, followed by incubation in a 37°C incubator for 20–24 h. Afterward, cells were stained with 0.1% crystal violet. The invaded cells were counted under an inverted optical microscope.

### ChIP assay

GBM cells were fixed to produce DNA-protein crosslinks. Then, cells were sonicated to produce chromatin fragments of an appropriate size. Lysates were then reacted with magnetic protein A beads with antibodies. Anti-HIF1α (ab2185, 1: 100, Abcam) and the NC IgG (rabbit, ab109489, 1: 100, Abcam) antibodies were used, followed by incubation with Protein Agarose/Sepharose. Finally, the precipitated SPOP (Forward: 5′-AATGAGCCTGTTGCTCCTGT-3′; Reverse: 5′-GGGCAACACCAGATGAGAAT-3′) was analyzed by RT-qPCR [44].

### Construction of a mouse model of GBM

Thirty SPF BALB/c nude mice (4–6 weeks; 16–20 g) were housed in an SPF laboratory (60–65% humidity, 22–25°C, a 12-h light/dark cycle) individually for one week, with free access to water and food. The mice were acclimatized for one week before experiment. The health of the mice was observed before the experiment.

The T98G cell suspension (1 × 10^7^ cells/mL) harboring NC-agomir + oe-NC, miR-873-5p agomir + oe-NC or miR-873-5p agomir + oe-SPOP (10 nmol/mouse, 100 μL, once 5 days, for 5 weeks) was subcutaneously inoculated into the left armpit of mice by 1 mL syringe. All overexpression vectors were constructed in the EcoRI site of the pLV vector. All agomir and overexpression plasmids were purchased from GenePharma. After 5 weeks, mice were euthanized, with the tumor removed and weighed.

### Statistical analysis

Measurement data, presented as mean ± standard deviation, are analyzed using SPSS 19.0 software (IBM Corp. Armonk, NY, USA). Data between two groups were compared by unpaired *t*-test. One-way ANOVA with Tukey’s test was applied for multi-group data comparison. Bonferroni-corrected two-way ANOVA or repeated measures ANOVA was employed for multi-group data comparison at varied time points. *p* < 0.05 was considered significant.

### Data availability statement

The data underlying this article will be shared on reasonable request to the corresponding author.

## Supplementary Materials

Supplementary Tables
