# Supplementary material for: Tumor-suppressive function and mechanism of miR-873-5p in glioblastoma: evidence based on bioinformatics analysis and experimental validation
Source: Aging (Albany NY). 2023 Jun 28;15(12):5412–25. doi: 10.18632/aging.204800 (PMC10333085; doi:10.18632/aging.204800)
Supplement: Supplementary Tables [file aging-15-204800-s001.pdf]

SUPPLEMENTARY TABLES

Supplementary Table 1. Sequences of cell transfection.

Item	Sequence (5′–3′)
NC mimic	GACGAUGACGUAGCGAUGAC
NC inhibitor	AUGCGACGCGAUGCAGUAGUC
miR-873-5p mimic	GCAGGAACUUGUGAGUCUCCU
miR-873-5p inhibitor	AGGAGACUCACAAGUUCCUGC
sh-NC	ACGTAGCAGTAGACGTCCGACCGAAGTCGGACGTCTACTGCTACGT
sh-HMOX1-1	GGGTGATAGAAGAGGCCAAGACGAATCTTGGCCTCTTCTATCACCC
sh-HMOX1-2	GCTCAACATCCAGCTCTTTGACGAATCAAAGAGCTGGATGTTGAGC
sh-HIF1α-1	GGAAATGAGAGAAATGCTTACCGAAGTAAGCATTTCTCTCATTTCC
sh-HIF1α-2	GCCGAGGAAGAACTATGAACACGAATGTTCATAGTTCTTCCTCGGC
sh-SPOP	GGTGCTACACACAGATCAAGGCGAACCTTGATCTGTGTGTAGCACC

Supplementary Table 2. Primer sequences for the RT-qPCR.

Target	Primer sequence (5′–3′)
miR-873-5p	Forward: 5′-GCAGGAACTTGTGAGTCTCCT-3'<br>Reverse universal primer
HMOX1	Forward: 5′-AGTCTTCGCCCCTGTCTACT-3′<br>Reverse: 5′-CTTCACATAGCGCTGCATGG-3′
SPOP	Forward: 5′-CAAGGCAAAGACTGGG-3′<br>Reverse: 5′-AACACTCACCTCGCAGA-3′
GAPDH	Forward: 5′-TGGGTGTGAACCATGAGAAG-3′<br>Reverse: 5′-GCTAAGCAGTTGGTGGTGC-3′
HIF1-α	Forward: 5′-GGCGCGAACGACAAGAAAAA-3′<br>Reverse: 5′-GTGGCAACTGATGAGCAAGC-3′
U6	Forward: 5′-GCTTCGGCAGCACATATACT-3′<br>Reverse universal primer

Supplementary Table 3. Primary antibodies used for Western blot.

Antibody	Product No.	Brand	City	Country
HMOX1	ab52947	Abcam	Cambridge	UK
HIF1α	ab51608	Abcam	Cambridge	UK
SPOP	ab137537	Abcam	Cambridge	UK
CCND1	ab16663	Abcam	Cambridge	UK
MYC	ab32072	Abcam	Cambridge	UK
MMP-7	ab5706	Abcam	Cambridge	UK
E-cadherin	ab40772	Abcam	Cambridge	UK
GAPDH	ab9485	Abcam	Cambridge	UK
N-cadherin	ab76011	Abcam	Cambridge	UK
